# Poor Lymphocyte Infiltration to Primary Tumors in Acral Lentiginous Melanoma and Mucosal Melanoma Compared to Cutaneous Melanoma

**DOI:** 10.3389/fonc.2020.524700

**Published:** 2020-12-17

**Authors:** Yoshiyuki Nakamura, Zhu Zhenjie, Kazumasa Oya, Ryota Tanaka, Yosuke Ishitsuka, Naoko Okiyama, Rei Watanabe, Yasuhiro Fujisawa

**Affiliations:** Department of Dermatology, Faculty of Medicine, University of Tsukuba, Tsukuba, Japan

**Keywords:** cutaneous, melanoma, acral lentiginous melanoma, mucosal melanoma, lymphocytes, checkpoint inhibitors

## Abstract

Recent clinical trials have demonstrated the efficacy of immune checkpoint inhibitors (ICIs) for treating melanoma. However, these previous studies comprised mainly Caucasian populations, in which cutaneous melanoma (CM) is the major clinical type. In contrast, Asian populations have a distinct profile of melanoma and show much higher frequencies of acral lentiginous melanoma (ALM) and mucosal melanoma (MCM). Compared with CM, ALM and MCM show poorer response to ICIs, but the mechanisms have not been fully understood. To evaluate the immune status in each melanoma subtype, we examined the number of total tumor-infiltrating lymphocytes (TILs), CD4^+^ TILs, CD8^+^ TILs, and tumor-infiltrating FoxP3^+^ regulatory T cells (Tregs) to evaluate the immune status in each melanoma subtype using data from 137 patients with melanoma. Total TIL numbers in ALM and MCM were significantly lower than that in CM. CD4^+^ TIL number in MCM was also lower than CM although CD4^+^ TIL number in ALM was comparable with CM. In contrast, CD8^+^ TIL numbers in both ALM and MCM were significantly lower than that in CM. Although number of tumor-infiltrating Tregs was comparable among the 3 subtypes, the proportion of tumor-infiltrating Tregs in CD4^+^ T cells in MCM was significantly higher than in CM and ALM. Multivariate regression analysis revealed that ALM and MCM were significantly associated with a lower total TIL number, but only MCM was significantly associated with a lower CD4^+^ TIL number. Multivariate regression analysis also revealed that both ALM and MCM were significantly associated with a lower CD8^+^ TIL number. Our results suggest that both ALM and MCM are independent factors of lower total TIL number, which may be associated with poorer responses to ICIs in ALM and MCM.

## Introduction

Malignant melanoma is an aggressive malignant tumor with high mortality (1). However, recent clinical trials have demonstrated the efficacy of immune checkpoint inhibitors (ICIs) for treating malignant melanoma. Both anti-programmed death-1 (PD-1) monoclonal antibodies (nivolumab and pembrolizumab) and anti-cytotoxic T-lymphocyte-associated antigen-4 (CTLA-4) monoclonal antibodies (ipilimumab) have been reported to prolong the overall survival in advanced melanoma patients (1–3). In addition, combination therapy of nivolumab plus ipilimumab showed better overall survival than ipilimumab alone (4). However, these previous studies comprised mainly Caucasian populations, in which cutaneous melanoma (CM) is the major clinical type of melanoma (5). In contrast, Asian populations reveal a distinct profile of melanoma from that of Caucasians with much higher frequencies of acral lentiginous melanoma (ALM) and mucosal melanoma (MCM). We previously showed that, compared with CM, ALM and MCM have a poorer response to ICIs (6, 7). However, the mechanisms underlying this poor response in ALM an MCM have not been fully elucidated.

PD-1 is an inhibitory receptor expressed mainly by activated T cells. CTLA-4 suppresses T cell activation through competing with CD28 in binding to CD80/86. Both anti-PD-1 antibodies and anti-CTLA-4 antibodies exert anti-tumor effects through T cell activation. Immune cells, including T cells, that infiltrate tumors may induce tumor regression following ICI treatment, and consistently, the number of tumor-infiltrating lymphocytes (TILs) have been reported to predict the tumor response to ICIs (8). Therefore, we speculate that TIL number may be lower in ALM and MCM than in CM. In this study, we examined number of total TILs, CD4^+^ TILs, CD8^+^ TILs, and tumor-infiltrating FoxP3^+^ regulatory T cells (Tregs) of the primary tumors for each melanoma subtype to evaluate the immune status.

## Material and Methods

### Patients

Data of patients with melanoma, who were referred to our department and whose primary tumors were resected at the University of Tsukuba Hospital from January 2004 to November 2019, were retrospectively collected. Diagnosis was confirmed using the pathological findings. Clinical factors including age, sex, melanoma subtype, tumor thickness, presence or absence of ulceration of primary tumors were collected. This study was approved by the institutional ethics committee of the University of Tsukuba Hospital. All the protocols in this study were performed in accordance with the Declaration of Helsinki.

### Immunohistochemical Studies

The specimens were fixed in formalin and embedded in paraffin. They were sliced into 3-µm thicknesses, mounted on glass slides, deparaffinized in xylene, and rehydrated before antigen retrieval by boiling in citrate buffer (0.01M citrate containing 0.5% Tween 20, pH 6.0). The sections were incubated in 10% bovine serum albumin in PBS containing 0.01% Tween 20 at room temperature for 1 hour and with optimized concentrations for 60 min at room temperature. The primary antibodies used in this experiment were antibodies for human CD4 (M7310, 1:250 dilution; Dako), CD8 (M7103, 1:250 dilution; Dako) and FoxP3 (ab20034, 1:200 dilution; Abcam) overnight at 4°C, followed by biotinylated anti-rat IgG antibody (1:500; Vector Laboratories) and VECTASTATIN ABC reagent (Vector Laboratories) at room temperature for 60 and 30 min, respectively. Finally, the sections were stained with DAB Peroxidase Substrate Kit (Vector Laboratories) before imaging. The number of positive cells was counted in a ×400 microscopic field (high power fields: HPF). Six areas were counted in each case, and the numbers were averaged. Total TIL number was calculated as the total number of CD4^+^ TILs plus CD8^+^ TILs. In addition to TIL number, we also evaluated the numbers of total T cells, CD4^+^ T cells, CD8^+^ T cells, and FoxP^+^ regulatory T cells (Tregs) in peritumoral skin/mucosa and distant normal skin/mucosa as controls. The peritumoral skin/mucosa and normal skin/mucosa were defined as areas at a distance of 0.3–2 mm and more than 4 mm from the lesions, respectively.

### Statistical Analysis

Kruskal-Wallis statistic, Mann-Whitney U test, and Chi square were used to compare clinical factors among melanoma subtypes. Univariate regression analyses were used to reveal the association of numbers of total TILs, CD4^+^ TILs or CD8^+^ TILs with clinical factors. For the factors shown to be significant in the univariate analysis, multivariate analyses in a stepwise procedure were performed to determine the independent factors associated with number of total TILs, CD4^+^ TILs or CD8^+^ TILs. In the procedure, a 0.15 significance level for entering and eliminating explanatory variables was used. Throughout the analyses, *P*-values of <0.05 were considered as statically significant. The statistical tests were 2-sided and carried out using Stat Flex version 6.0 (Artec, Osaka, Japan) and Prim version 6 (Graph Pad software, California, USA).

## Results

### Patient Background

We collected data for 137 patients with melanoma (**Table 1**). The mean age was 68.4 (range: 19 to 93) and 63 patients (46.0%) were male. The most common melanoma type was ALM (65 patients, 47.4%), followed by CM (53 patients, 38.7%), and MCM (19 patients, 13.9%). Among the 19 MCM patients, 17 (89.4%) were referred to our department after the 2014 introduction of ICIs to Japan. Twenty-four patients (36.9%) with ungual melanoma were included in the ALM category. Primary tumor locations in the patients with MCM included: genital lesions (5 patients), conjunctiva lesions (5 patients), nasal cavity (4 patients), oral cavity (2 patients), anorectal lesion (2 patients), and esophageal lesion (1 patient). The mean tumor thickness was 4.7mm (range: 0.2 to 22) and ulceration was present in 72 patients (52.6%). There were 61 patients at stage I/II, 28 at stage III, and 48 at stage IV. The mean number of total TILs, CD4^+^ TILs, CD8^+^ TILs and tumor-infiltrating Tregs were 59.3/HPF (range: 3.7 to 151.3), 22.2/HPF (range: 1.0 to 64.7), 37.2/HPF (range: 2.7 to 101.3) and 7.3/HPF (range: 0.8 to 19.7), respectively. The mean number of total T cells, CD4^+^ T cells, CD8^+^ T cells and Tregs in peritumoral skin/mucosa were 22.1/HPF (range: 10.3 to 45.5), 11.1/HPF (range: 1.3 to 28.7), 11.9/HPF (range: 3.2 to 30.2) and 4.0/HPF (range: 0.3 to 10.8), respectively. The mean number of total T cells, CD4^+^ T cells, CD8^+^ T cells and Tregs in distant normal skin/mucosa were 9.9/HPF (range: 2.0 to 54.2), 5.5/HPF (range: 0.8 to 23.1), 4.4/HPF (range: 0.8 to 26.2) and 1.0/HPF (range: 0.2 to 6.7), respectively. The occurrence of distant normal skin/mucosa in 4 patients (1 with ALM patient and 3 with MCM) was not included in the resected specimens, and was excluded for the analysis.

**Table 1:** Patients’ background and data for each melanoma subtype.

Clinical factors	All subtypes	CM	ALM	MCM	*P*-value
Age					0.028
Mean (range)	68.4 (19–93)	64.0 (19–93)	71.5 (36–93)	70.4 (52–80)	
Sex					0.12
Male	63	23	33	7	
Female	74	30	32	12	
Tumor thickness					0.30
Mean (range)	4.7(0.2–22)	5.0 (0.5–19)	4.1 (0.4–17)	5.6 (0.2–22)	
Ulceration					0.022
Presence	72	20	40	12	
Absence	65	33	25	7	
Stage					0.0097
I/II	61	26	30	5	
III	28	9	18	1	
IV	48	18	17	13	
Total TIL number/HFP					0.0008
Mean (range)	59.3 (3.7–151.3),	72.9 (9.0–151.3)	54.2 (7.5–146.7)	39.5 (3.7–88.5)	
CD4^+^ TIL number/HPF					0.030
Mean (range)	22.2 (1.0–64.7)	26.3 (1.5–66)	21.2 (1.5–51.5)	14.1 (1.0–40.8)	
CD8^+^ TIL number/HPF					0.0004
Mean (range)	37.2 (2.7–101.3)	46.5 (6.2–101.3)	33.0 (3.7–90.7)	25.4 (2.7–46.8)	
Tumor-infiltrating Tregs/HPF					0.467
Mean (range)	7.3 (0.8–19.7)	7.4 (0.8–18.0)	7.7 (0.8–19.7)	6.2 (0.8–13.3)	

CM, cutaneous melanoma; ALM, acral lentiginous melanoma; MCM, mucosal melanoma; TIL, tumor-infiltrating lymphocyte; Tregs, regulatory T cells; HPF, high power field. P-value was calculated using Kruskal-Wallis statistic or Chi square test.

### Comparison of Clinical Factors Among Melanoma Subtypes

**Figure 1:**
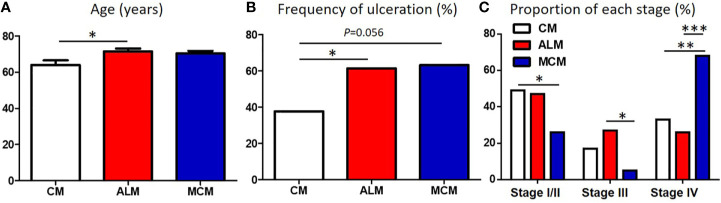
Comparison of patients’ background between each melanoma subtype. **(A–C)** Age **(A)**, frequency of ulceration **(B)** and proportion of each stage **(C)** in patients with cutaneous melanoma (CM), acral lentiginous melanoma (ALM) and mucosal melanoma (MCM) were shown. *P*-values was calculated using Mann-Whitney U test **(A)** and Chi square test **(B, C)**. *P<0.05, **<0.01, ***P<0.001.

**Figure 2:**
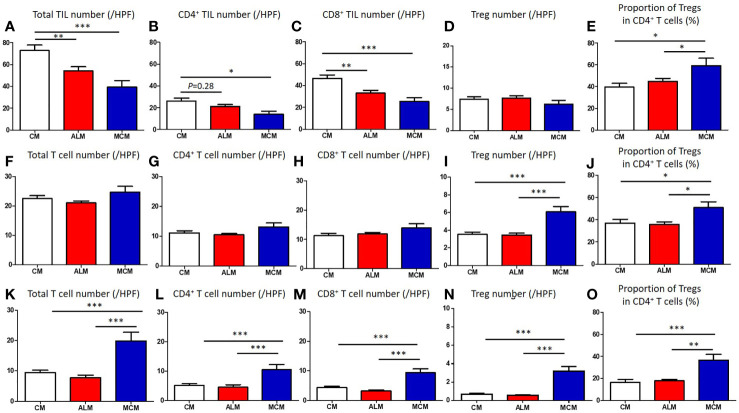
Comparison of T cells in tumor, peritumoral skin/mucosa and distant normal skin/mucosa of each melanoma subtype. **(A–O)** Numbers of total T cells, CD4^+^ T cells, CD8^+^ T cells, regulatory T cells (Tregs) and proportion of Tregs in CD4^+^ T cells in tumor **(A–E)**, peritumoral skin/mucosa **(F–J)** and distant normal skin/mucosa **(K–O)** in patients with cutaneous melanoma (CM), acral lentiginous melanoma (ALM) and mucosal melanoma (MCM) were shown. *P*-values was calculated using Mann-Whitney U test. *P<0.05, **<0.01, ***P<0.001.

We compared the clinical factors among the melanoma subtypes and found that 2 factors, sex and tumor thickness, were comparable among the subtypes (**Table 1**). In contrast, patients with ALM were significantly older than those with CM (**Table 1**, **Figure 1A**). Frequency of ulceration in ALM was significantly higher than CM, and MCM also tended to show higher frequency than CM (**Table 1**, **Figure 1B**). The proportion of patients at stage I/II in CM and at stage III in ALM were significantly higher than those in MCM (**Table 1**, **Figure 1C**). In contrast, the proportions of patients at stage IV in CM and ALM were significantly lower than in MCM (**Table 1**, **Figure 1C**). Total TIL numbers in ALM and MCM were significantly lower than in CM (**Figure 2A**). CD4^+^ TIL number in MCM was also lower than in CM, although CD4^+^ TIL number in ALM was comparable with CM (**Figure 2B**, **Supplemental Figures S1A–C**). In contrast, CD8^+^ TIL numbers in both ALM and MCM were significantly lower than in CM (**Figure 2C**, **Supplemental Figures S1D–F**). Although the number of tumor-infiltrating Tregs was comparable among the 3 subtypes, the proportion of tumor-infiltrating Tregs in CD4^+^ T cells in MCM was significantly higher than in CM and ALM (**Figures 2D, E**, **Supplemental Figure S1G–I**). In peritumoral skin/mucosa, both number of Tregs and proportion of Tregs in CD4^+^ T cells were significantly higher in MCM than CM and ALM, although the numbers of total T cells, CD4^+^ T cells, and CD8^+^ T cells were comparable among the 3 subtypes (**Figures 2F–J**, **Supplemental Figures S2A–C**). In the normal skin/mucosa, the number of total T cells, CD4^+^ T cells, CD8^+^ T cells, Tregs, and proportion of Tregs in CD4^+^ T cells in MCM were all significantly higher than in CM and ALM (**Figures 2K-O**, **Supplemental Figures S2D–F**).

### Associations of TIL Number With Clinical Factors

Univariate regression analysis showed that MCM was significantly associated with lower total TIL number, and that ALM and tumor thickness showed a tendency toward lower and higher numbers of total TILs, respectively (**Table 2**). Multivariate analysis revealed that ALM and MCM were significantly associated with lower total TIL number (**Table 2**), suggesting that both ALM and MCM were independent factors of lower total TIL number. As for CD4^+^ TILs, univariate regression analysis showed that only MCM was significantly associated with lower CD4^+^ TIL number, and the significance was retained in multivariate analysis (**Table 3**). In contrast, univariate regression analysis showed that both ALM and MCM were significantly associated with lower numbers of CD8^+^ TILs, and tumor thickness showed a tendency towards higher numbers of CD8^+^ TILs (**Table 4**). The significance of both ALM and MCM was also retained in multivariate analysis (**Table 4**).

**Table 2:** Association of total TIL number with clinical factors.

Clinical factors	Univariate regression analysis				Multivariate regression analysis			
	Estimate	SE	t-value	*P*-value	Estimate	SE	t-value	*P*-value
Age(continuous value)	−0.147	0.194	0.759	0.449				
Sex (Ref: female)	1.340	5.942	0.226	0.822				
Clinical type (Ref: CM)								
ALM	−9.844	5.864	1.679	0.0955	−17.519	6.015	2.913	0.0042
MCM	−23.091	8.326	2.773	0.0063	−34.168	8.659	3.946	0.0001
Tumor thickness (continuous value)	1.263	0.689	1.833	0.0690	1.261	0.658	1.918	0.0573
Ulceration	0.257	5.924	0.0434	0.965				
Stage (Ref: stage I/II)								
Stage III	6.065	7.318	0.829	0.409				
Stage IV	−1.186	6.20	0.191	0.849				

CM, cutaneous melanoma; ALM, acral lentiginous melanoma; MCM, mucosal melanoma; TIL, tumor-infiltrating lymphocyte; SE, standard error.

**Table 3:** Association of CD4^+^ TIL number with clinical factors.

Clinical factors	Univariate regression analysis				Multivariate regression analysis			
	Estimate	SE	t-value	*P*-value	Estimate	SE	t-value	*P*-value
Age (continuous value)	−0.0544	0.0925	0.588	0.558				
Sex (Ref: female)	−0.5633	2.827	0.199	0.842				
Clinical type (Ref: CM)								
ALM	−1.931	2.813	0.686	0.494	−4.692	2.964	1.583	0.116
MCM	−9.361	3.991	2.346	0.0205	−12.523	4.267	2.935	0.0039
Tumor thickness (continuous value)	0.486	0.329	1.476	0.142	0.511	0.324	1.577	0.117
Ulceration	0.761	2.817	0.270	0.788				
Stage (Ref: stage I/II)								
Stage III	1.521	3.487	0.436	0.663				
Stage IV	1.060	2.948	0.360	0.720				

CM, cutaneous melanoma; ALM, acral lentiginous melanoma; MCM, mucosal melanoma; TIL, tumor-infiltrating lymphocyte; SE, standard error.

**Table 4:** Association of CD8^+^ TIL number with clinical factors.

Clinical factors	Univariate regression analysis				Multivariate regression analysis			
	Estimate	SE	t-value	*P*-value	Estimate	SE	t-value	*P*-value
Age (continuous value)	−0.0931	0.129	0.723	0.471				
Sex (Ref: female)	1.904	3.933	0.484	0.629				
Clinical type (Ref: CM)								
ALM	−7.914	3.865	2.048	0.0425	−12.827	3.991	3.214	0.0016
MCM	−13.730	5.545	2.476	0.0145	−21.645	5.745	3.768	0.0002
Tumor thickness (continuous value)	0.777	0.457	1.699	0.0915	0.751	0.436	1.720	0.0878
Ulceration	−0.503	3.924	0.128	0.898				
Stage (Ref: stage I/II)								
Stage III	4.544	4.84	0.938	0.350				
Stage IV	−2.246	4.103	0.547	0.585				

CM, cutaneous melanoma; ALM, acral lentiginous melanoma; MCM, mucosal melanoma; TIL, tumor-infiltrating lymphocyte; SE, standard error.

## Discussion

Previously, Wada et al. demonstrated that melanoma-specific survival in patients with ALM was comparable to those with CM when treated with dacarbazine-based chemotherapies before introduction of ICIs. They proposed that ALM does not differ in its biological behavior from CM (9). In contrast, it has been reported that ALM and MCM were less susceptible to ICI treatment than CM (6, 10). In addition, Maeda et al. demonstrated that overall survival and progression survival in patients with ALM and MCM tended to be shorter than those with CM when treated with nivolumab monotherapy, suggesting that the immune response to ALM and MCM may be poorer than CM (10). However, only a few reports have shown the comparison of basic immune status between each melanoma subtype (11).

Our study revealed that total TIL number in ALM and MCM were both significantly lower than in CM, and multivariate regression analysis showed ALM and MCM to be independent factors of lower total TIL number. The exact mechanism underlying the lower number of TILs in ALM and MCM remains unknown. However, given that ALM and MCM have been reported to show lower mutation burden than CM, the low mutation burden, which results in poor generation of tumor neo-antigen, may be involved in the poor proliferation of tumor-specific T cells in ALM and MCM (12).

Recently, Castaneda et al. demonstrated that the number of TILs in ALM was lower than that in CM, which is consistent with the findings in our study. However, we discovered that, in ALM, while the number of CD8^+^ TIL was significantly lower than in CM the number of CD4^+^ TILs was not, suggesting that lower number of TILs in ALM may be a result of the lower numbers of CD8^+^ TILs. Previous studies reported that there are differences in the molecules responsible for migration, retention, and maintenance between CD4^+^ T cells and CD8^+^ T cells (13–15). Therefore, the distinct expression patterns of these molecules associated with migration, retention, and maintenance in ALM and CM may have led to the finding that the number of CD8^+^ TILs, but not CD4^+^ TILs, in ALM was significantly lower than in CM, although the exact mechanism remains unknown.

Both CD4^+^ T cells and CD8^+^ T cells play crucial roles in the immune response to tumors. However, CD4^+^ T cells, including Tregs, may also exhibit significant immunosuppressive function for tumors (16, 17). In contrast, CD8^+^ TILs have been shown to predict favorable prognoses in many types of cancer (18, 19). Additionally, it has been reported that a high ratio of total TIL number to CD4^+^ TIL number predicted better survival in patients with bladder cancer (20). Moreover, anti-PD-1 antibody was totally dependent on CD8^+^ T cells to exert anti-tumor immunity in the mouse colon cancer model (21). Therefore, we speculate that poor infiltration of CD8^+^ T cells, but not CD4^+^ T cells, may play an important role in limiting the response of ALM to ICI treatment.

In MCM, the numbers of both CD4^+^ TILs and CD8^+^ TILs were significantly lower than those in CM. The digestive system manifests immune tolerance to a large variety of antigens, including dietary proteins and commensal bacteria (22). This mechanism within the digestive system includes enriched immune suppressive cells such as Tregs and inhibitory cytokines such as TGF-β and IL-10 (22). In addition, conjunctiva and genital tracts have been reported to show immune tolerance, which may also involve Tregs (23–25). Indeed, in our study, both the number of Tregs and proportion of Tregs in CD4^+^ T cells were significantly higher in MCM than in CM and ALM in both the peritumoral skin/mucosa and distant normal skin/mucosa. In addition, the proportion of tumor-infiltrating Tregs in CD4^+^ T cells in MCM was also significantly higher than in CM and ALM. Therefore, basic immune tolerance associated with mucosal site may also contribute the low number of both CD4^+^ TILs and CD8^+^ TILs in MCM observed in our study.

In conclusion, our study demonstrated that the number of TILs in ALM and MCM were significantly lower than CM, which may be associated with poorer responses to ICIs in ALM and MCM. Therefore, in addition to ICIs, novel therapies to increase the number of TILs are required for enhancing the tumor immune response in ALM and MCM. Our study was limited by the relatively low number of participants, especially MCM patients. In our study, only 2 of 19 patients with MCM (10.6%) were referred to our department before the introduction of ICIs. In addition, the proportion of patients at stage IV in MCM was significantly higher than in CM and ALM. Therefore, a substantial number of MCM patients who were not indicated for the recently-developed systemic therapies including ICIs may have been treated in other departments or other institutions, resulting in the low number of MCM patients in our study. Therefore, further large-scale studies with a larger number of MCM patients are needed to confirm our findings.

## Data Availability Statement

The original contributions presented in the study are included in the article/**Supplementary Material**. Further inquiries can be directed to the corresponding author.

## Author Contributions

YN mainly conducted the research, analyzed the data, and wrote the manuscript. ZZ, KO, and RT supported the immunohistochemical studies. YI, NO, RW, and YF provided the helpful suggestions for the research. All authors contributed to the article and approved the submitted version.

## Conflict of Interest

The authors declare that the research was conducted in the absence of any commercial or financial relationships that could be construed as a potential conflict of interest.

## Supplementary Material

Supplementary Figure 1 Immunohistochemical studies for CD4, CD8 and FoxP3 in tumor of each melanoma subtype. **(A–I)** Representative images of immunohistologic analyses of CD4 in the tumor of cutaneous melanoma (CM) **(A)**, acral lentiginous melanoma (ALM) **(B)**, and mucosal melanoma (MCM) **(C)**, CD8 in the tumor of CM **(D)**, ALM **(E)**, and MCM **(F)**, and FoxP3 in the tumor of CM **(G)**, ALM **(H)**, and MCM **(I)** (×200, respectively). Scale bar, 50 μm.

Supplementary Figure 2 Immunohistochemical studies for FoxP3 in peritumoral skin/mucosa and distant normal skin/mucosa of each melanoma subtype. Representative images of immunohistologic analyses of FoxP3 in the peritumoral skin/mucosa of cutaneous melanoma (CM) **(A)**, acral lentiginous melanoma (ALM) **(B)**, and mucosal melanoma (MCM) **(C)**, and in the distant normal skin/mucosa of CM **(D)**, ALM **(E)**, and MCM **(F)** (×200, respectively). Scale bar, 50 μm.
