# Supplementary figures and images for: Poor Lymphocyte Infiltration to Primary Tumors in Acral Lentiginous Melanoma and Mucosal Melanoma Compared to Cutaneous Melanoma
Source: Front Oncol. 2020 Dec 17;10:524700. doi: 10.3389/fonc.2020.524700 (PMC7773936; doi:10.3389/fonc.2020.524700)

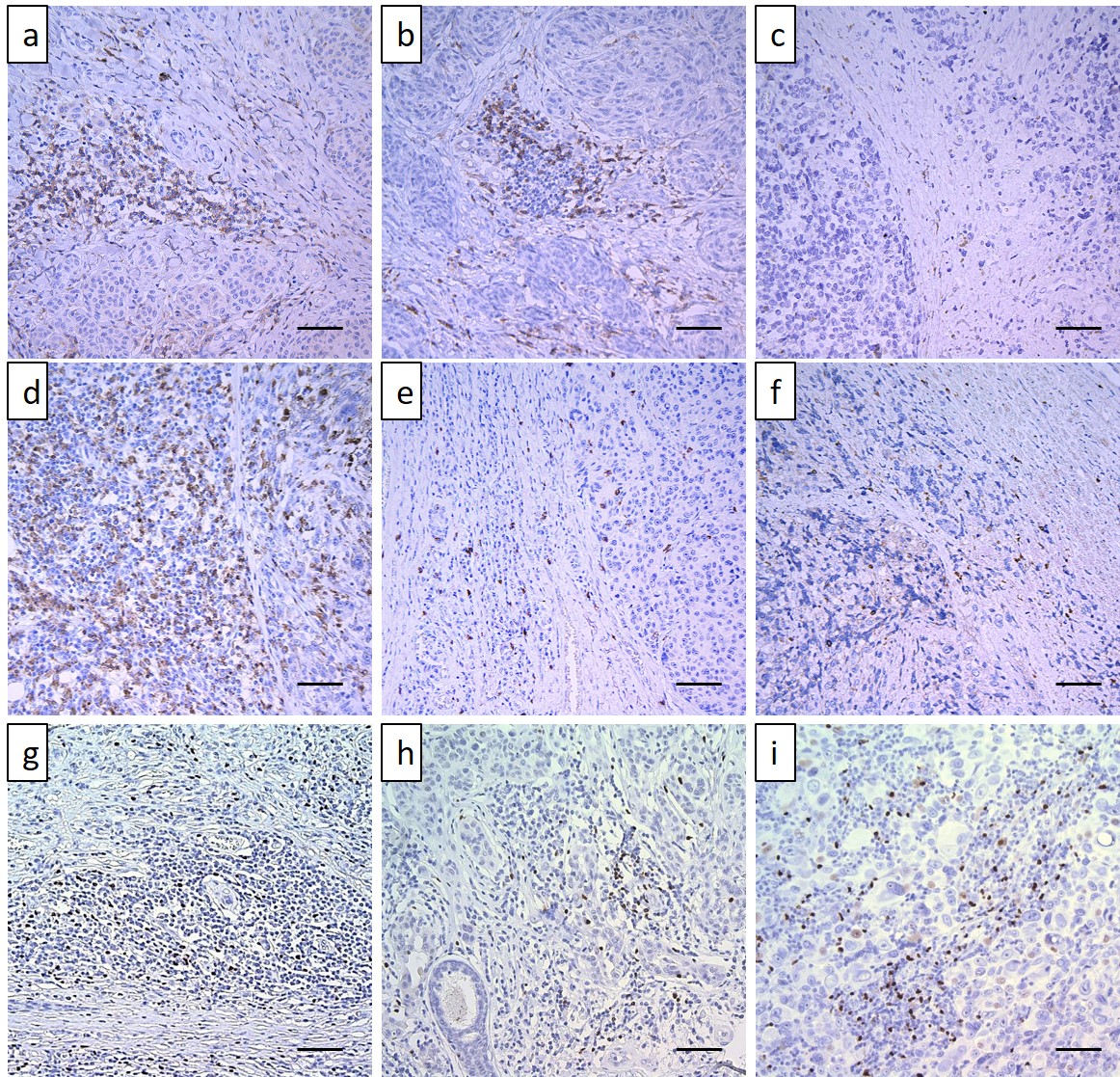

Supplement: Supplementary Figure 1 — Immunohistochemical studies for CD4, CD8 and FoxP3 in tumor of each melanoma subtype. (A–I) Representative images of immunohistologic analyses of CD4 in the tumor of cutaneous melanoma (CM) (A), acral lentiginous melanoma (ALM) (B), and mucosal melanoma (MCM) (C), CD8 in the tumor of CM (D), ALM (E), and MCM (F), and FoxP3 in the tumor of CM (G), ALM (H), and MCM (I) (×200, respectively). Scale bar, 50 μm. [file Image_1.jpeg]

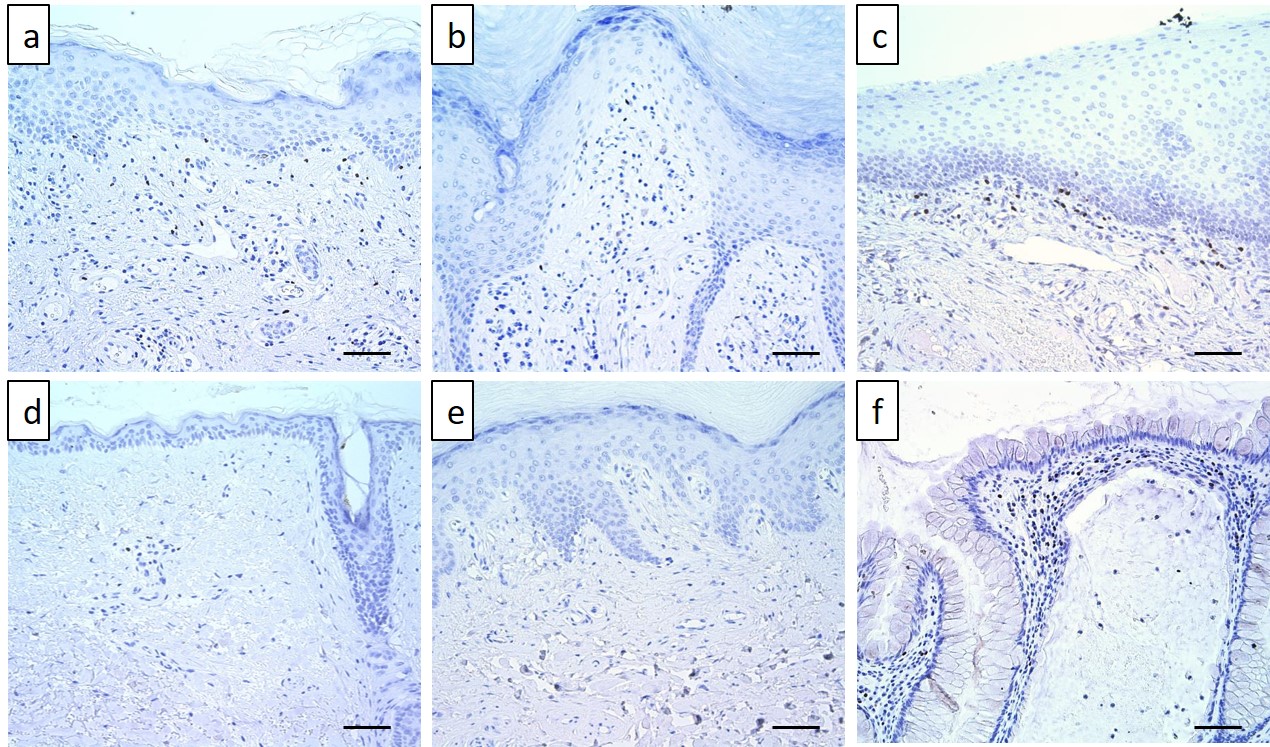

Supplement: Supplementary Figure 2 — Immunohistochemical studies for FoxP3 in peritumoral skin/mucosa and distant normal skin/mucosa of each melanoma subtype. Representative images of immunohistologic analyses of FoxP3 in the peritumoral skin/mucosa of cutaneous melanoma (CM) (A), acral lentiginous melanoma (ALM) (B), and mucosal melanoma (MCM) (C), and in the distant normal skin/mucosa of CM (D), ALM (E), and MCM (F) (×200, respectively). Scale bar, 50 μm. [file Image_2.jpeg]
